# GnRH antagonist versus agonist in normoresponders undergoing ICSI: a randomized clinical trial in Iran

**Published:** 2011

**Authors:** Ensieh Tehraninejad, Akram Ghahghaei Nezamabadi, Batool Rashidi, Maryam Sohrabi, Maryam Bagheri, Fedyeh Haghollahi, Elham Azimi Nekoo, Mina Jafarabadi

**Affiliations:** Reproductive Health Research Center, Tehran University of Medical Sciences, Tehran, Iran.

**Keywords:** *IVF*, *GnRH agonist*, *GnRH antagonist*, *Normoresponder*

## Abstract

**Background:**

General concern is that the pregnancy rate is higher with GnRH-agonist as a protocol of pituitary suppression. GnRH-antagonist protocol provides a shorter period of administration and an easy flexible protocol.

**Objective:**

In this study, the outcomes of GnRH agonist and antagonist in ICSI cycles are compared in normo responder patients.

**Materials and Methods:**

In this randomized clinical trial, 300 normoresponders undergoing ICSI were randomly divided to GnRh agonist (n=150) and GnRh antagonist (n=150) groups. The main outcome measurements were chemical, clinical and ongoing pregnancy rates (PR).

**Results:**

The mean duration of stimulation were 9.6±1.6 and 8.2±1.6 days in agonist and antagonist groups respectively (p=0.001). The mean number of MII oocyte retrieved in agonist and antagonist groups were 7.7±4.0 and 6.9±4.3 respectively (p=0.03). There was no significant difference between two groups regarding mean number of gonadotrophin ampoules, follicles, occytes, total embryos and good quality embryos, OHSS incidence, and abortion rate. Chemical pregnancy rate was 35.3% in agonist and 39.3% in antagonist group. Clinical pregnancy rate was 35.3% in agonist and 34% in antagonist group. Ongoing pregnancy rate was 45 (31.3%) in agonist and 44 (29.3%) in antagonist group. There was no significant difference between two groups in pregnancy rates.

**Conclusion:**

In this study antagonist protocol was shown to be an easy, safe and friendly protocol in Iranian normoresponder patients, having similar outcomes with standard agonist protocol but shorter period of stimulation.

## Introduction

The first in vitro fertilization (IVF) therapy was performed in a natural cycle. Gonadothropins are given to induce multiple follicular development and GnRH analogues are used for the prevention of premature LH surges in IVF. LH surges occur in about 20% of stimulated IVF patients (1). Preventing LH surges using GnRH analogues improves oocyte yielded with more embryos, allowing better selection and leading to an increase in pregnancy rate. 

GnRH agonist administration causes gonadotrophin suppression via pituitary desensitization, after an initial short period of gonadotrophin hypersecretion. In contrast, GnRH antagonist accuses immediate and rapid gonadotrophin suppression by competitive occupancy of GnRH receptor and therefore is a choice to use in IVF for the prevention of premature LH surge (2). 

Several potential advantages of antagonists are suggested over GnRH agonists. Among these advantages are shorter duration of injectable drug treatment, decreased gonadotropin requirement per cycle, and lower overall treatment cost (3). Although agonist use is accompanied by a series of disadvantages including hypoestrogenemia, cyst formation, requirement for a prolonged period of down-regulation and an increase in FSH and LH in primary administration, agonist protocol was well accepted in clinical practice, and general concern is that the pregnancy rate was higher with agonist protocol (4, 5). 

The recent development of side- effect free GnRH-antagonist protocol, with immediate blockage of receptors and shorter period of administration, provides physicians with an easy flexible protocol and offers patients a side-effect free, “friendly” protocol (4). Comparative studies between GnRH analogues in IVF cycles have suggested that the duration of stimulation in the antagonist group was shorter with lower incidence of OHSS, but in several outcomes the results of studies remain controversial (6-11). 

Several studies are done in different subgroups of patients to recognize the best protocol of pituitary suppression (11- 15). The aim of this study was to compare outcomes of GnRH agonist and antagonist stimulation protocols and to evaluate the potential benefits of GnRH antagonist utilization in ART cycles in normoresponder Iranian patients. Normo-responder hints the group of patients with neither decreased ovarian reserve nor predisposition to hyperstimulation. The study was approved by ethics committee of Tehran University of Medical Sciences.

## Materials and methods

This randomized clinical trial was conducted at Vali-e-Asr Reproductive Health Research Center and Rooyan Institute, Tehran, Iran from January 2008 to January 2010. In total 300 patients undergoing ICSI cycles with or without ICSI were evaluated in this study. After obtaining informed consent, patients were allocated to two groups according to a sequence of computer generated random numbers (0 or 1). 

A total of 300 women were randomized, 150 in each group. Inclusion criteria were: age<38 years, normal basal serum FSH, 20≤ BMI< 30kg/ m^2^ and regular menstrual cycle. Exclusion criteria were: PCOS, severe endometriosis, history of poor response in previous treatment cycles and history of repeated IVF failure (more than 3 failed cycles). The primary outcome measures were fertilization and pregnancy rates. Additional outcomes of interest were number of oocytes retrieved, number of good quality embryos transferred, OHSS incidence and patient's capacitance.

In the agonist group on cycle day 21, Busereline acetate (Superfact, Aventis, Germany) was started as 0.5 mg daily subcutaneous (S.C.) injection until menstruation had begun and adequate suppression was achieved (serum estradiol level <50 pg/ml and no ovarian cystic structures on ultrasound examination). At day 3 of next menstrual cycle, the dose of Busereline was diminished to 0.2 mg and rFSH (Gonal F, Merck Serono, Switzerland) was started. The starting dose for the first 5 days varied between 150-225 IU daily by S.C. injection depending on the age (< or >35 years) and history of patient. Thereafter, transvaginal ultrasonography was done every other day and the dose was adjusted on the basis of follicle graph using Gonal-F and HMG (Menoupour, Ferring, USA). Ovulation was induced with 10000 IU, IM injection of HCG (Profasi, Serono, Switzerland) when at least 2 follicles 18-20 mm were observed and serum estradiol was between 1000 and 3000 pg/ml. In the antagonist group, rFSH treatment was begun on day 3 of menstrual cycle. The starting dose for the first 5 days varied between 150-225 IU S.C. depending on the patient's age and history. Thereafter transvaginal ultrasonography was done every other day and the dose was adjusted on the basis of follicle graph using Gonal-F and HMG. When there was one follicle 14mm in diameter, antagonist (Cetrotide, Merck Serono, Germany), 0.25 mg S.C. daily dose was administrated until the day of HCG administration. The time of cetrotide injection was adjusted not to be more than 30 hours apart from HCG administration. When at least 2 follicles 18-20 mm in diameter were seen, rFSH and HCG (10000 IU, IM) was injected. Oocyte retrieval was performed 36h after HCG administration, by transvaginal sonography guided puncture of follicles. Two or three embryos were transferred 72 hours after oocyte retrieval using Cook catheter (Cook Medical Incorporated, Bloomington, USA). 

In both groups the luteal phase was supported with vaginal suppository of cycleogest 400 mg/BD. Progesterone treatment was started on the day of oocyte retrieval and continued until the day of pregnancy test performed 14 days after the embryo transfer. In the case of a positive test, this regiment was continued during the first trimester of pregnancy. Clinical pregnancy was defined as the presence of a gestational sac with visible heartbeat. 

Embryos were scored based on the assessment of the number and distribution of nucleoli precursor bodies in the pronucleus to have good and poor morphology (16). The OHSS classification utilized in this study was the one proposed by Golan *et al* (17). 

**Statistical analysis**

All analyses were performed using SPSS (version 16) with a two-sided 5% significance level.

## Results

In this study, 150 patients treated with agonist protocol were compared with 150 patients treated with the antagonist protocol. Two groups were matched regarding age, BMI, duration of infertility, cause of infertility, number of pervious attempts and baseline FSH (Table I). Two groups showed no significant difference regarding mean number of gonadotropin ampoules used (p=0.63), mean number of follicles ≥15mm on oocyte retrieval day (p=0.12) and mean number of oocytes retrieved (p=0.31) (Table II). Chemical, clinical and ongoing pregnancy rates in two groups were not significantly different (p=0.42, 0.83 and 0.71 respectively) (Table II).

There was no significant difference between two groups regarding mean number of good quality embryos (p=0.50), abortion rate (p=0.09) and incidence of OHSS (p= 0.25) (Table II). 

The duration of stimulation in agonist group was significantly higher than antagonist group (9.6±1.6 vs. 8.2±1.6 days, p=0.00).

This study showed significant difference between two groups regarding endometrial thickness on the day of HCC administration (10.3mm in agonist vs. 9.3 mm in antagonist group, p= 0.00). Mean number of M II oocytes retrieved in agonist group was also significantly higher than antagonist group (7.7±4 vs. 6.9±4.3, p=0.03). 

**Table I:** Demographic and clinical characteristics of patients in two groups

**Characteristic**	**GnRH agonist protocol (n=150)**	**GnRH antagonist protocol (n=150)**	**p-value**
Age (yrs)	30.4	31.1	0.52
BMI (kg/m^2^)	26.7	24.7	0.41
Duration of infertility (yrs)	7.8	7.6	0.61
No. of previous attempts	0.95	0.85	0.09
Baseline FSH (mlU/mL)	6.4±1.0	6.5±1.2	0.06
Cause of infertility Female factor [n (%)] Male factor [n (%)] Male and female [n (%)] Unexplained [n (%)]	52 (34%) 70 (46%) 14 (9%) 14 (9%)	54 (36%) 68 (45%) 16 (10%) 12 (8%)	0.71 0.34 0.34 0.51

**Table II:** Clinical and laboratory outcomes in two groups

	**GnRH agonist (n=150)**	**GnRH antagonist (n=150)**	**p-value**
Duration of stimulation (Day, Mean± SD)	9.6±1.6	8.2±1.6	0.00
Number of gonadotropin ampoules (Mean±SD)	24.2±7.3	24.2±6.5	0.63
Number of mature follicle (Mean±SD)	11.3±6.3	10.7±6.6	0.12
Number of oocyte retrieved (Mean±SD)	9.2±4.2	8.6±4.3	0.31
Number of MII oocyte (Mean±SD)	7.7±4.0	6.9±4.3	0.03
Number of embryo (Mean±SD)	5.3±3.4	5.6±3.6	0.50
Number of good quality embryo (Mean±SD)	4±2.4	3.9±2.4	0.81
Chemical pregnancy rate (n %)	53 (35.3%)	59 (39.3%)	0.43
Clinical pregnancy rate (n %)	53 (35.3%)	51 (34%)	0.80
Ongoing pregnancy rate (n %)	47 (31.3%)	44 (29.3%)	0.72
Abortion (n %)	9 (17%)	18 (30%)	0.09
OHSS (n %)	26 (17.3%)	19 (12.7%)	0.25
Endometrial thickness (mm) (Mean±SD)	10.3±1.3	9.3±1.3	0.00
Mean cost of one cycle prior to oocyte retrieval (visit+ sonography+ medication) (million Rials)	6.16 ± 0.23	5.90 ± 0.47	0.07

## Discussion

In this study the results of GnRH antagonist multiple doses protocol usage were compared versus long protocol of GnRH agonist in ICSI cycles in Iranian normoresponder patients. 

Apart from significantly higher number of MII oocyte in agonist group and shorter duration of stimulation in antagonist group in our study there was no difference in the number of follicles, total retrieved oocytes, total embryos, good quality embryos and mean cost of one cycle prior to oocyte retrieval between two groups and as the main outcome measurement the rates of chemical, clinical and ongoing pregnancy were similar in two groups. 

Despite the result of some studies confirming our results (9, 17-21), in meta-analysis of 5 RCTs Aboulghar and Al-Inany reported that clinical pregnancy rate was 5% lower in antagonist protocol (5). In present study, the mean duration of stimulation days was significantly longer in agonist group. Many studies are in accordance with it (Greco *et al*: 11.1±0.3 vs. 12.2±0.4, Kumbak *et al*: 11.7±1.2 vs. 12.9±1.6 and Xavier *et al*: 9.5±1.7 vs. 10.6±2.1) (17, 21, 22). 

In present study, the mean dose of gonadotropin used in two protocols had no significant difference and this was similar to the result of the studies of Berger (19), Xavier (18), Kumback (23) and Kolibiakis meta-analysis (24). Although some other researches reported the total dose of gonadotrophin used in the agonist protocol was significantly higher than antagonist protocol (10, 22). No sever OHSS occurred in either group during our study and the incidence of mild OHSS was higher in agonist protocol but this was not statistically significant. We excluded PCOS patients from the study and this can be interpreted as the cause of absence of severs OHSS in our study. 

Using antagonist protocol for preventing OHSS especially in PCOS patients is proved in several studies (12-15). In present study the rate of abortion was higher in antagonist group but this was not statistically significant (p=0.09). Bahceci *et al*, 2009, reported that the rate of early pregnancy loss (EPL) was higher in antagonist protocol (26). 

The endometrial thickness in the antagonist protocol was lower than agonist in the day of hCG administration (10.3 mm vs. 9.3mm, p=0.00) in our study. Xavier *et al* reported that there wasn’t any significant difference between 2 protocols in this variable (18) but Orveito *et al* reported that endometrial receptivity and endometrial thickness was higher in the agonist protocol (27).

GnRH antagonist molecules are potent inhibitors of cell cycle, decreasing the synthesis of locally produced growth factors. They can exhibit this activity in all tissues presenting GnRH receptors and consequently influence blastomere formation, endometrium development and fulliculogenesis and oocyte maturation (22). This can explain the lower number of MII occyte and lower endometrial thickness in antagonist protocol and may reflect the cause of slight (but not significant) increase in abortion rate in antagonist group in present study. 

The results of this study show that these two protocols are very similar in outcomes in normoresponder patients. Immediate mode of action, flexibility of use, shorter duration of administration, shorter duration of FSH stimulation, and a lower incidence of hospital admission due to sever OHSS make the antagonist protocol an excellent approach for ovarian stimulation in IVF. There was no significant difference in the rate of live birth in GnRH antagonist protocol comparing with agonist in the study accomplished by Kolibinakis and Tarletzis in 2006 (24). Literature suggests that the side effect, physiologic and psychological distress and treatment burden is lower in antagonist protocol (28), though these points were not concerned in present study and is proposed to be evaluated in further studies in Iranian patients.

On the basis of the results of this RCT on Iranian normoresponder women, we offer using the “GnRH Antagonist” as a patient friendly protocol for the first choice in ART cycle with lower incidence of side effects, similar pregnancy rate and cost and time saving. 

## Note

***Registretion ID in IRCT: IRCT138902283950N1***
